# Diagnostic utility of BINAX NOW RSV – an evaluation of the diagnostic performance of BINAX NOW RSV in comparison with cell culture and direct immunofluorescence

**DOI:** 10.1186/1476-0711-5-13

**Published:** 2006-06-06

**Authors:** Nteimam Jonathan

**Affiliations:** 1 Health Protection Agency, Birmingham Heartlands Hospital, Bordesley Green East, Birmingham, B9 5SS, UK

## Abstract

**Background:**

The regular increase in the incidence of respiratory illness caused by respiratory syncytial virus (RSV) during winter months in the United Kingdom, and other countries with temperate climate is usually accompanied by increased bed pressures especially in paediatric units in these countries. As a result, there is usually an increase in the demand for infection control services during these months. This makes obvious the need for making a rapid diagnosis of the infection during these months. BINAX NOW RSV (Maine, USA), a rapid membrane based immunochromatographic assay was designed to achieve this objective.

**Methods:**

This study evaluated the diagnostic performance of BINAX NOW RSV in comparison with the methods routinely used in our laboratory namely direct immunofluorescence (DIF) and cell culture.

**Results and conclusion:**

Results indicate that Binax Now RSV could be relied on to make infection control decisions in paediatric units during periods of peak RSV activity.

## Background

Respiratory syncytial virus (RSV) is the most common cause of bronchiolitis and pneumonia among infants and children under 1 year of age. In temperate climates, RSV infections usually occur during annual community outbreaks, often lasting 4 to 6 months, during the late fall, winter, or early spring months. The timing and severity of outbreaks in a community vary from year to year. RSV spreads efficiently among children during the annual outbreaks, and most children will have serologic evidence of RSV infection by 2 years of age[1].

Virus isolation is considered the 'gold standard' method of RSV diagnosis. However it is cumbersome and may also require up to a week to produce a result. The shell vial method which essentially involves centrifugation of the specimen directly onto the cell monolayer at 700 g for 1 hour may reduce this time considerably to about 24 hours[2,3]. For optimal recovery of the virus by cell culture, it is essential that specimen be transported to the laboratory at refrigerator temperature (4°C) or on wet ice[4]. If it is anticipated that samples may take longer than 24 hours between collection and cell inoculation then the specimen should be rapidly frozen to -70°C to maintain viable viral titre[4]. A combination of human epithelial cell lines, primary monkey kidney cell lines and human fibroblasts is necessary for optimal recovery of the virus[4].

Direct Immunofluorescence (DIF) is a quicker method of achieving RSV antigen detection in respiratory specimens, making it possible to have a result a few hours after specimen collection. However it is a subjective test and could give false positive results[5]. Furthermore, it is a labour intensive technique, takes longer to perform than the rapid test and requires well trained, experienced technologists for correct interpretation of results.

NOW RSV (Binax, Maine USA) is a rapid membrane based immunochromatographic assay which is FDA approved for the qualitative detection of RSV fusion proteins in nasal wash and nasopharyngeal specimens from symptomatic children less than 5 years of age[6]. The test takes 15 minutes to perform.

Using this test method a result could be achieved within 2 hours or less of specimen collection.

The current testing protocol in our laboratory for the diagnosis of respiratory viral infection is to carry out DIF and cell culture on all nasopharyngeal aspirates and broncho-alveolar lavages. During the winter months when the incidence of RSV infection increases, it becomes necessary to rapidly make a diagnosis for prompt institution of infection control measures especially in paediatric wards to limit the spread of the infection. NOW RSV appears to be promising in achieving this objective.

## Objective

The aim of this study was to assess the diagnostic performance of NOW RSV in comparison with tissue culture and DIF.

## Materials and methods

100 (one hundred) specimens consisting of 91 nasopharyngeal aspirates (NPA) and 9 broncho-alveolar lavages (BAL) received in the virus isolation unit of the West Midlands Public Health Laboratory (Health Protection Agency), Birmingham Heartlands Hospital between October and December 2003 were tested in parallel by Now RSV, DIF and cell culture for evidence of RSV infection. All the BAL specimens were from adult patients (age range 19 – 69 years). 53 NPAs were from infants <5 months old, 27 from children aged 5 months to 5 years and 11 from older children and adults (age range 13 – 52 years).

### NOW RSV

Binax NOW RSV is a rapid membrane based immunochromatographic technique which is designed to detect RSV fusion protein antigen in nasal washes and nasopharyngeal swab specimens. The test utilises anti RSV antibody conjugated to visualising particles and adsorbed onto a nitrocellulose membrane to form the 'sample line'. Performing the test involves a one step technique of adding the sample to the white pad at the top of the test strip, and incubating for 15 minutes. There is no need for any special laboratory equipment, and no need for any special incubation conditions. The specimens were tested, and results read according to the manufacturer's instruction [6].

### Virus isolation

About 1 ml of the specimen was added to 2 ml of Virus Transport Medium (VTM) and mixed. VTM contains a salt solution to ensure proper ionic concentration, a buffer to maintain ph, a source of protein for viral particle stability, antibiotics and antifungal to prevent bacterial and fungal overgrowth [7]. A few drops were then inoculated into healthy monolayers of MRC5, RMK and PLC (Primary liver carcinoma) cell lines (ECACC Porton Down UK). Inoculated tubes were incubated in rotary drums at 33°C and examined twice a week for cytopathological effect (CPE). When a significant CPE was detected, the culture medium was discarded leaving a few drops into which the cells on the culture tube were scrapped. The suspended cells were spotted onto teflon coated slides previously cleaned with alcohol and treated as for DIF (see below). Specimens that showed no CPE after 7 days of incubation were subjected to haemadsorption. Immunofluorescence was carried out on positive haemadsorbing specimens. Specimens which gave a negative reaction to haemadsorption were incubated for a further 7 days. Specimens which showed no CPE after 2 weeks of incubation were discarded and reported as negative after a negative haemadsorption test.

### Direct immunofluorescence

Specimens in the universal container were centrifuged at low speed for 10 minutes. The supernatant was discarded and 10 mls of PBS (phosphate buffered saline) was added to the deposit, mixed well and re-centrifuged at the same speed. A third centrifugation step was done if the original specimen contained a lot of mucus. After discarding the supernatant, the cell deposit was re-suspended in a few drops of PBS and spotted onto teflon coated slides previously cleaned with alcohol, allowed to dry, fixed in acetone for 10 minutes and stained with anti RSV monoclonal antibody conjugated with fluorescein isothiocyanate (FITC) (IMAGEN DAKO, Cambridgeshire UK). Slides were incubated at 37°C for 30 minutes in a humid chamber, rinsed, and washed in PBS for 5 minutes. Slides were then drained dry, mounted with a cover slip using mounting fluid (IMAGEN, DAKO Cambridgeshire, UK) and examined by fluorescent microscope.

A sample was considered true positive if it was a) positive by cell culture or b) positive by DIF and NOW RSV, if culture is negative. A true negative result was defined as a negative result obtained by cell culture, and at least one of the other two methods.

## Results

11 out of the 100 specimens (11%) were positive for RSV by cell culture, 23% (23 out of 100) were positive by DIF while 15% (15 out of 100) were positive by NOW RSV (Table 1). 19% (19 out of 100) were considered true positives – 11 positive by cell culture and 8 positive by both NOW RSV and DIF. 81% (81 out of 100) were negative by culture and at least one other method (true negatives). 7 specimens were positive by all three methods.

**Table 1:** Overall results by BINAX NOW RSV, DIF and cell culture

	NOW RSV	DIF	CELL CULTURE
POSITIVE	15	23	11
NEGATIVE	85	77	89
TOTAL	100	100	100

Using the above definitions of true positive and true negative respectively, the sensitivity and specificity of NOW RSV, DIF and cell culture respectively were 15/19(78.9%) and 81/81(100%); 19/19(100%) and 77/81(95.1%); 11/19(58.9%) and 81/81(100%).

Out of the 91 NPAs 75 were negative either by cell culture or by both NOW RSV and DIF, (true negatives), 16 were positive either by cell culture or by the other two methods (true positives). 14 of these true positive specimens were positive by NOW RSV giving a sensitivity of 87.5%. All 75 true negative specimens were negative by NOW RSV giving a specificity of 100%. Similarly, DIF and cell culture had sensitivity and specificity respectively of 100%(16/16) and 94.7%(71/75); and 43.8%(7/16) and 100%(75/75) respectively.

Of the 9 BAL specimens, 6 were negative by all three test methods, 1 was positive by all three methods, and 2 were positive by DIF and cell culture, and negative by NOW RSV. Thus sensitivities and specificities for NOW RSV, DIF and cell culture were 33.3% (1/3) and 100% (6/6); 100% (3/3) and 100% (6/6); 100% (3/3) and 100% (6/6) respectively (table 3). One of the 6 negative BAL specimens was found positive for HSV 1 by DIF and cell culture.

All 11 NPAs from older children and adult (13 – 52 years) were negative for RSV by all three test methods. 5 of these specimens were found positive for influenza A virus by DIF and cell culture.

Of the 27 NPAs from children aged 5 months to 5 years, 20 were negative by all three test methods, 2 were positive by all three methods, 2 were positive by NOW RSV and DIF, while 3 were positive by only DIF. Thus sensitivities and specificities in this age group for NOW RSV, DIF and cell culture were 100% (4/4) and 100% (23/23); 100% (4/4) and 87% (20/23) and 50% (2/4) and 100% (23/23) respectively (table 3). 7 out of the 23 true negative specimens were found positive for influenza A by DIF and cell culture.

Out of the 53 NPAs from infants aged less than 5 months, 12 were positive by either cell culture or NOW RSV and DIF (true positives) and 41 were negative by cell culture and at least one other method (true negatives). 1 specimen was positive by DIF but negative by NOW RSV and cell culture. 10 out of the 12 true positive specimens were positive by NOW RSV, and all 41 true negative specimens were found negative by that test method. All 12 true positive specimens were found positive by DIF while 40 out of the 41 true negative specimens were found negative by DIF. 6 out of the 12 true positive specimens were found positive by cell culture, and all 41 true negative specimens were found negative by cell culture. Thus sensitivities and specificities for NOW RSV, DIF and cell culture were 83% (10/12) and 100% (41/41); 100% (12/12) and 97.6% (40/41); 50% (6/12) and 100% (41/41) respectively (Table 2). 5 out of the negative specimens in this group were positive for influenza A by DIF and cell culture, 1 specimen was positive for parainfluenza virus 1 and 2 were positive for parainfluenza virus 2. The specimen which gave a positive result for RSV by DIF only was also found positive for enterovirus (untyped) by cell culture.

**Table 2:** Test performance on 53 NPAs from patients less than 5 months old.

	NOW RSV	DIF	CELL CULTURE
TRUE POSITIVE	10	12	6
FALSE POSITIVE	0	1	0
TRUE NEGATIVE	41	40	41
FALSE NEGATIVE	2	0	6
TOTAL	53	53	53
SENSITIVITY (%)	83	100	50
SPECIFICITY (%)	100	97.6	100

**Table 3:** Summary of the test performance of the three methods.

SPECIMEN TYPE	TEST METHOD	TEST PERFORMANCE			
		Sensitivity (%)	Specificity (%)	PPV* (%)	NPV+ (%)

NPA <5 months (n = 53)	NOW RSV	10/12 (83)	41/41 (100)	100	95.35
	DIF	12/12 (100)	40/41 (97.6)	92.31	100
	CELL CULTURE	6/12 (50)	41/41 (100)	100	87.23
NPA 5 months – 5 years (n = 27)	NOW RSV	4/4 (100)	23/23 (100)	100	100
	DIF	4/4 (100)	20/23 (87)	57.14	100
	CELL CULTURE	4/4 (50)	23/23 (100)	100	92
BAL Adults (n = 9)	NOW RSV	1/3 (33.3)	6/6 (100)	100	75
	DIF	3/3 (100)	6/6 (100)	100	100
	CELL CULTURE	3/3 (100)	6/6 (100)	100	100

*PPV = Positive predictive value

+NPV = Negative predictive value

A summary of the three test methods performance on the different specimens tested is presented in Table 3.

## Discussion

The ease of performance and the rapidity of result reporting make NOW RSV test method a very useful tool especially in the winter months when the incidence of RSV infection rises. This is especially useful in making decisions on instituting infection control measures in paediatric units. The high specificity of this test method indicates that it could be relied on in making such decisions. The reduced sensitivity of 78.9% (overall) or 87.5% (NPAs) found in this study re-echoes the manufacturer's warning on limitations of the assay. It is recommended that a negative specimen be tested by another sensitive rapid method like direct immunofluorescence to improve sensitivity.

In a similar evaluation conducted by Mackie PL et al[8], on the use of NOW RSV as a point of care test (POCT) for the diagnosis of RSV, sensitivity and specificity values of 87% and 94% respectively were obtained when compared to direct immunofluorescence alone. These values increased to 92% and 94% respectively when discrepant results were re-tested by laboratory personnel according to the manufacturer's instructions. In this study, using the three test methods of NOW RSV, DIF and virus isolation, and defining a true positive as a positive result obtained by cell culture or both NOW RSV and DIF if culture is negative; and a true negative as a negative result obtained by cell culture and at least one other method; DIF correctly identified all the positive samples but also had the highest number of false positive results. On the other hand all the samples identified as positive by NOW RSV were true positives. 4 samples (overall) or 2 samples (NPAs) found negative by NOW RSV were actually positive. Low viral load in such specimens may possibly account for the false negative results obtained [6].

The relatively poor performance of virus isolation in terms of sensitivity may be explained at least in part by non-viability of Viral particles in the specimens, when they were received in the laboratory. Virus isolation was the only test method out of the three that required the presence of viable viral particles to achieve a positive result. A significant number of these specimens were from hospitals or laboratories located in different cities from our laboratory, and some of these specimens were not received in the laboratory until the following day. Furthermore, these specimens were not transported to the laboratory at optimal temperatures (none of these samples were shipped in cold boxes or on ice packs). Considerable delay was also noted from the time of sample collection from in patients at the Birmingham Heartlands Hospital and reception at our laboratory where the specimens were tested. Further delay may possibly have occurred from the time specimen was received at the central specimen processing unit to when the specimen actually got to the virus isolation laboratory.

The poor performance of NOW RSV on BAL specimens compared to DIF and cell culture may be worthy of note. However final conclusions can not be drawn from this study as the number of samples tested was small. In a similar study conducted by Ohm-Smith et al[5], sensitivity and specificity values found for DIF and NOW RSV were 93% and 97%; 89% and 100% respectively, using cell culture as the gold standard. A reduced sensitivity of NOW RSV on respiratory samples from adults was demonstrated by their findings. Their tests were conducted on fresh samples only. In this study, none of the 11 nasopharyngeal specimens tested from older children (>13 years) and adults gave a positive result by any of the three test methods. This finding is in consonance with the fact that immunity to RSV is usually acquired early in life [1]. About half of the specimens in this age group were found positive for influenza A virus, indicating that influenza A virus remains the leading cause of respiratory disease in adulthood during winter.

## Conclusion and recommendation

The absolute specificity of NOW RSV found in this study indicates that this rapid test method could be relied on to make infection control decisions during periods of peak RSV activity. The need to adhere to the manufacturer's advice to subject samples which give negative results to another test method is also being emphasized. Virus isolation might probably retain it's position as the final arbiter in RSV diagnosis not only because of it's absolute specificity as indicated in this study but also because it remains the only test method that makes available viable viral particles for further laboratory studies.

Finally, it could be said from the results of this evaluation study, that the combination NOW RSV and DIF is likely to yield reliable results in the rapid diagnosis of respiratory infections caused by RSV.

## References

[B1] Gregory StorchA, Gregory A Storch Respiratory infections. Essentials of Diagnostic Virology.

[B2] Engler HD, Preuss J (1997). Laboratory diagnosis of Respiratory Syncytial Virus Infection in 24 hours of utilising shell vial cultures. J Clin Microbiol.

[B3] Leland DS, Edwin H, Lennette, Thomas F, Smith (1999). Clinical Virology: Concepts and Perspectives. Laboratory Diagnosis of Viral infections.

[B4] Tristram DA, Welliver RC, Edwin H, Lennette, Thomas F, Smith (1999). Respiratory Syncytial Virus. Laboratory Diagnosis of Viral infections.

[B5] Ohm-Smith MJ, Nassos PS, Haller BL (2004). Evaluation of the Binax NOW, BD Directigen, and BD Directigen EZ assays for detection of respiratory syncytial virus. J Clin Microbiol.

[B6] (1998). Binax inc. Portland, Maine USA. NOW® RSV Test kit Technical instructions booklet.

[B7] Richard Buller, Gregory A Storch (2000). Specimen collection and transport. Essentials of Diagnostic Virology.

[B8] Mackie PL, McCormick EL, Williams C (2004). Evaluation of Binax NOW® RSV as an acute point -of-care screening test in a paediatric accident and emergency unit. Commun Dis Public Health.

